# Prevalence and Determinants of the Composite Index of Anthropometric Failure in Children Under Five Years in Bangladesh: Insights From a National Survey

**DOI:** 10.1002/fsn3.70499

**Published:** 2025-06-22

**Authors:** Rafid Hassan, Shahadoth Hossain, Md Mahbub Alam, Sanjib Saha, Tanjina Akter, Masum Ali, Md Ruhul Amin

**Affiliations:** ^1^ Nutrition Research Division International Centre for Diarrhoeal Disease Research, Bangladesh (icddr,b) Dhaka Bangladesh; ^2^ Institute of Nutrition and Food Science University of Dhaka Dhaka Bangladesh; ^3^ Health Economics Unit, Department of Clinical Science (Malmo) Lund University Lund Sweden; ^4^ Department of Zoology University of Dhaka Dhaka Bangladesh; ^5^ Nutrition and Health Science, Laney Graduate School Emory University Atlanta USA

**Keywords:** Bangladesh demographic and health survey, children, composite index of anthropometric failure, malnutrition, undernutrition

## Abstract

Child undernutrition remains a significant public health issue in Bangladesh, with high rates of stunting, wasting, and underweight. The Composite Index of Anthropometric Failure (CIAF) offers a comprehensive tool for assessing overall undernutrition. Although previous studies in Bangladesh have utilized CIAF, they have relied on older datasets. Estimating the current status is essential for designing and implementing targeted interventions due to the dynamic nature of undernutrition. This study aimed to provide updated insights by analyzing the CIAF and its determinants among children under 5 years, using the most recent 2022 Bangladesh Demographic and Health Survey (BDHS) data. A total of 3950 under‐5‐year‐old children were included. The modified Poisson regression was employed to identify the associated factors. The overall prevalence of CIAF was 33.7%, with subgroups of wasted and underweight (4.7%), stunted and underweight (10.8%), wasting, stunting, and underweight (3.4%), only wasted (3%), only stunted (8.8%), and only underweight (3%). Factors associated with higher risk of CIAF included older aged child, higher birth order (ARR: 1.30, 95% CI: 1.08–1.58), smaller birth size (ARR: 1.45, 95% CI: 1.24–1.70), uneducated mothers (ARR: 1.49, 95% CI: 1.16–1.93), maternal underweight (ARR: 1.28, 95% CI: 1.13–1.46), short stature (ARR: 1.54, 95% CI: 1.40–1.70). Children from the poorest households (ARR: 1.25, 95% CI: 1.02–1.53) and Sylhet (ARR: 1.22, 95% CI: 1.02–1.47) also had a higher risk. This study suggests strengthening community‐based nutrition‐specific programs along with improving access to healthcare, family planning, food assistance, and income opportunities to reduce child undernutrition in Bangladesh.

## Graphical abstract

Approximately one‐third of under five children in Bangladesh experienced anthropometric failure, including combinations such as wasted and underweight (4.7%), stunted and underweight (10.8%), all three conditions (3.4%), as well as only wasted (3%), only stunted (8.8%), and only underweight (3%). Key risk factors associated with CIAF include older child age, higher birth order, smaller birth size, maternal undernutrition and low education, poverty, and residence in Sylhet. Targeted community‐based nutrition interventions and improved access to healthcare and social support are recommended to address child undernutrition.

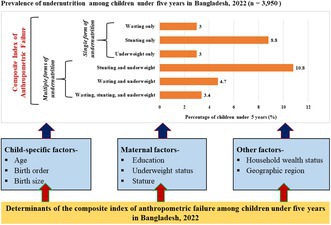

## 1 Introduction

Undernutrition among children under five is a significant public health issue. In 2022, worldwide approximately 148.1 million under‐five children were stunted, whereas 45 million suffered from wasting (UNICEF, WHO, and World Bank 2023). Each year, 4.9 million under‐five children die, predominantly in developing countries (UN IGME 2024), and almost half of these fatalities are associated with undernutrition (WHO 2024). These challenges are most prominent in Asia, where more than half of the world's stunted children and 70% of wasted children reside (UNICEF, WHO, and World Bank 2023). Child undernutrition delays physical and cognitive development, impairs academic performance, increases susceptibility to illness, and raises their risk of chronic disease later in life (Morales et al. 2023).

Bangladesh is undergoing rapid socioeconomic development with notable improvements in health and economic sectors (World Bank 2023). The country is facing a dual burden of disease—transitioning from communicable to non‐communicable diseases—and a nutritional shift from undernutrition to overnutrition (Rahman et al. 2019; Vatsa et al. 2023). Nevertheless, undernutrition remains a critical concern, particularly among children under five. According to the Bangladesh Demographic and Health Survey (BDHS) 2022, stunting, wasting, and underweight continue to afflict a large portion of Bangladeshi children (NIPORT & ICF 2024). In 2018, approximately 38% of children experienced anthropometric failure, with 11.3% facing severe failure (Chowdhury, Khan, et al. 2022; Kundu et al. 2022). Furthermore, 19.3% experienced multiple types of undernutrition, whereas 18.9% were impacted by a single type (Chowdhury, Khan, Rashid, et al. 2021).

Traditionally, undernutrition has been measured using solely conventional indicators like stunting, wasting, and underweight, which often overlook the overlap among these conditions (Nandy et al. 2005). However, in many Asian countries, including Bangladesh, many children suffer from overlapping or multiple forms of undernutrition (MFU) concurrently (Chowdhury, Khan, Rashid, et al. 2021; Kundu et al. 2022; Roy and Mondal 2024). Underweight children frequently experience stunting or wasting, with some exhibiting all three forms of anthropometric failure simultaneously (Chowdhury, Khan, Rashid, et al. 2021; Roy and Mondal 2024). Studies found that MFU accounted for 43% of child deaths (Gausman et al. 2021). Relying solely on conventional indicators may therefore underestimate the true burden of undernutrition. The Composite Index of Anthropometric Failure (CIAF) addresses this limitation by integrating stunting, wasting, and underweight into six mutually exclusive categories, providing a more holistic understanding of undernutrition (Nandy et al. 2005). Given the persistent burden of undernutrition in Bangladesh, employing the CIAF is crucial for accurately identifying vulnerable children and designing targeted nutritional interventions and policies.

Child undernutrition in Bangladesh is influenced by a complex interaction of various socio‐demographic, economic, environmental, and other contributing factors. It may arise from a combination of factors such as insufficient food intake, poor maternal health, limited access to quality healthcare, and inadequate infant feeding practices (Agabiirwe et al. 2022). Studies have shown that maternal characteristics (age, height, education, occupation, body mass index, decision‐making power) and paternal factors (education, occupation) significantly impact child nutrition (Chowdhury, Khan, Rashid, et al. 2021; Kundu et al. 2022; Shihab et al. 2022; Sumon et al. 2023). Furthermore, household characteristics, including food insecurity, place of residence, regional disparities, socioeconomic status, family size, and access to water, sanitation, and hygiene (WASH) facilities, play a crucial role (Bornee et al. 2025; Gebretsadik et al. 2023; Hasan et al. 2023; Rahman and Hossain 2022). Moreover, child‐specific factors such as age, sex, birth order, birth size, antenatal care visit, duration of breastfeeding, diet, health status, and history of illness have also been found to be associated with undernutrition (Chowdhury, Khan, Rashid, et al. 2021; Chowdhury, Rahman, et al. 2022; Gebretsadik et al. 2023; Islam and Biswas 2020; Kundu et al. 2022; Rahman et al. 2020).

Most studies on child undernutrition in Bangladesh have primarily focused on conventional indicators such as stunting, wasting, and underweight. Although a few studies have explored coexisting forms of malnutrition (Chowdhury, Khan, Rashid, et al. 2021; Chowdhury, Rahman, et al. 2022; Sumon et al. 2023) and the CIAF (Anik et al. 2021; Chowdhury, Khan, and Mondal 2021; Chowdhury, Khan, et al. 2022; Islam and Biswas 2020; Kundu et al. 2022), research using more recent data remains scarce. Previous studies identified the key factors influencing CIAF, including birth weight (Anik et al. 2021; Chowdhury, Khan, et al. 2022), birth order (Islam and Biswas 2020), maternal education (Chowdhury, Khan, and Mondal 2021; Kundu et al. 2022), and wealth of household (Chowdhury, Khan, and Mondal 2021), while birth weight and the age of the child emerged as the most influential factors for coexisting forms of malnutrition (Chowdhury, Khan, Rashid, et al. 2021; Chowdhury, Rahman, et al. 2022; Sumon et al. 2023). These studies highlighted changing patterns of factors influencing undernutrition in Bangladesh; however, they were based on earlier datasets. Malnutrition is a complex issue that varies over time. Understanding whether these determinants have remained consistent, increased, or decreased in influence over time is critical, especially in light of Bangladesh's ongoing rapid socioeconomic transformations. Therefore, this study utilized the latest BDHS‐2022 data to examine the prevalence and determinants of CIAF, providing updated insights into child undernutrition in the country.

## 2 Methods

### 2.1 Data Source

**FIGURE 1:**
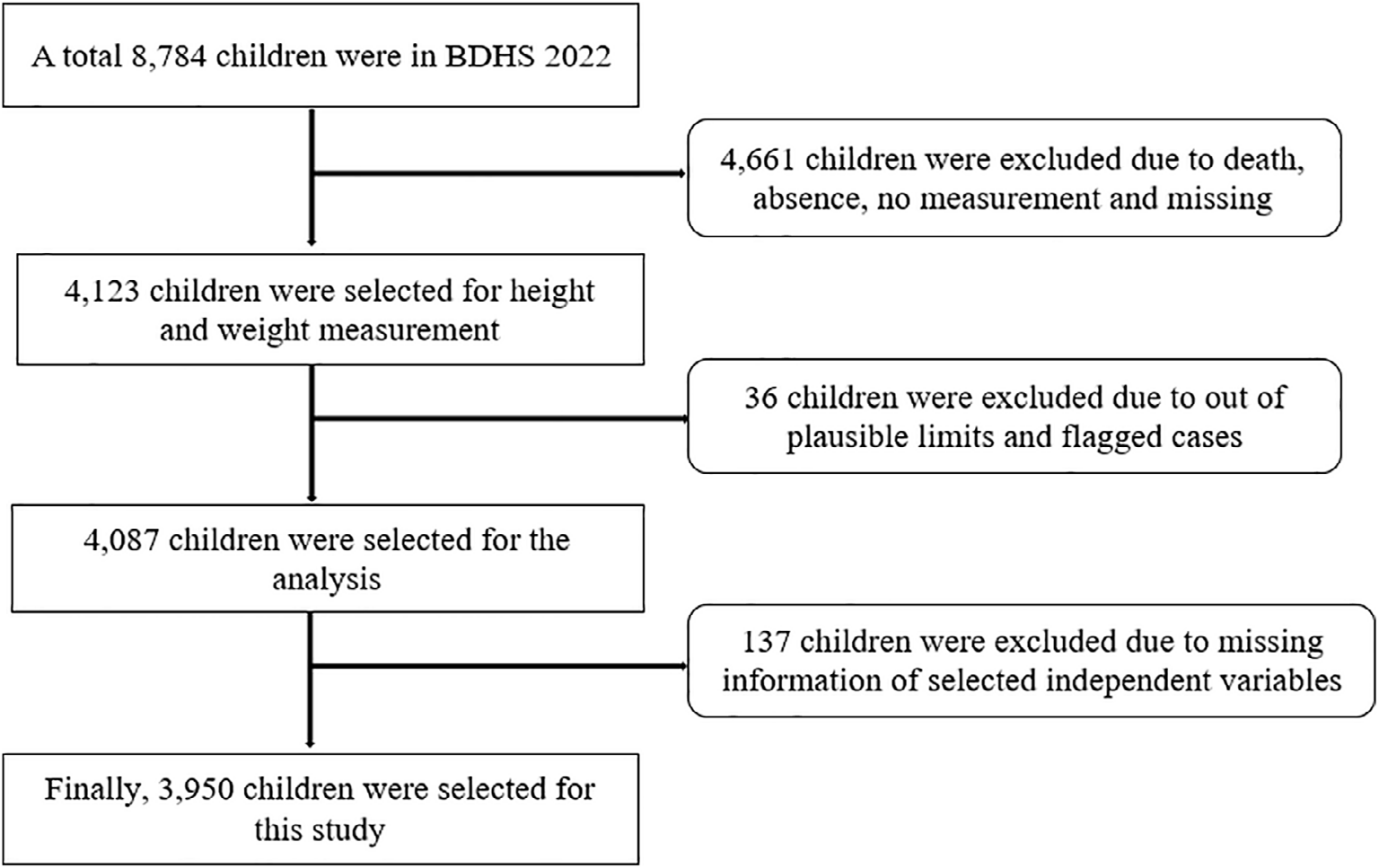
Schematic diagram of sample selection.

The study utilized a secondary dataset extracted from the BDHS‐2022. The survey employed a two‐stage stratified sampling method. In the first stage, 438 rural enumeration units and 237 urban enumeration units were selected using a probability proportional to size sampling technique. A complete list of all households within each selected enumeration unit was prepared during this phase. In the second stage, 30 households from each enumeration unit were selected using systematic sampling. The survey ultimately covered 8784 children across 675 clusters nationwide. After excluding cases with missing data, the final sample included 3950 children under the age of five (Figure 1). Details of the sampling procedure can be found elsewhere (NIPORT & ICF 2024).

### 2.2 Outcome Variable

In this study, the main outcome variable was CIAF. The CIAF was used to assess overall undernutrition, incorporating various combinations of child undernutrition: wasting only, stunting only, underweight only, wasting and underweight, underweight and stunting, or a combination of wasting, underweight, and stunting (Nandy et al. 2005). A child was classified as stunted, wasted, or underweight if their height‐for‐age, weight‐for‐height, or weight‐for‐age *z*‐score was below −2 standard deviations (SD) from the World Health Organization reference population median (WHO 2006). A child was classified as having multiple forms of undernutrition (MFU) if any combination of underweight and stunting, wasting and underweight, or all three conditions—wasting, underweight, and stunting was exhibited. In contrast, a child was considered to have a single form of undernutrition (SFU) if only one of these conditions: wasting, stunting, or underweight was experienced (Chowdhury, Khan, Rashid, et al. 2021). Detailed classification can be found in Table S1.

### 2.3 Independent Variables

The potential explanatory variables were selected based on the UNICEF conceptual framework (UNICEF 2021), the availability of the variables in the BDHS dataset, and earlier studies (Anik et al. 2021; Bornee et al. 2025; Chowdhury, Khan, and Mondal 2021; Chowdhury, Khan, Rashid, et al. 2021; Chowdhury, Khan, et al. 2022; Chowdhury, Rahman, et al. 2022; Islam and Biswas 2020; Kundu et al. 2022; Sumon et al. 2023).

Children characteristics included sex of children, age (0–11, 12–23, 24–35, 36–47, 48–59) in months, birth order (1st, 2nd, 3rd, ≥ 4), birth size (average or larger, smaller than average); maternal characteristics included age (15–19, 20–24, 25–34, ≥ 35) in years, occupation (currently working, currently not working), attitude toward wife‐beating (not justified, justified), decision‐making autonomy (practiced, not practiced), antenatal care (ANC) visit (≥ 4, < 4), stature (short, tall), maternal underweight (yes, no), exposed to media (exposed, not at all), both maternal and paternal education (no education, primary, secondary, higher); household characteristics included toilet facility (improved, unimproved), wealth index (poorest, poorer, middle, richer, richest), place of residence (urban, rural), region (Barisal, Chattogram, Dhaka, Khulna, Mymensingh, Rajshahi, Rangpur, Sylhet). Detailed definitions are in the Table S2.

### 2.4 Statistical Analysis

In this study, categorical variables were summarized using frequency and percentage. The Chi‐square test was conducted to examine the differences in the prevalence of the outcome variable across background characteristics. Factors associated with the CIAF were determined using modified Poisson regression. As logistic regression tends to overestimate associations when outcome prevalence exceeds 10% (Zhang and Yu 1998), log‐binomial or modified Poisson models were recommended alternatives (Chen et al. 2018). We used modified Poisson regression in this study since it provides unbiased estimates even under model misspecification (Zou 2004). Both unadjusted and adjusted risk ratios (RRs) with 95% confidence intervals (CIs) were reported. Variables with a *p* < 0.25 in the univariate analysis were included in the final multivariate model (Bursac et al. 2008). Multicollinearity was checked using the variance inflation factor (VIF), with a cutoff < 10 (Table S3). Statistical significance was set at *p* < 0.05, *p* < 0.01, and *p* < 0.001. All analyses accounted for clustering, stratification, and sampling weights. Missing data were excluded, and all analyses were conducted in STATA, version 15 (StataCorp, College Station, TX 77845, USA).

### 2.5 Ethics Statement

This study utilized secondary data from the Demographic and Health Survey (DHS). To access these data, a formal request, detailing the research objectives and purpose, was submitted to the Data Archivist of the DHS Program. Permission to access and use the BDHS datasets was granted for this research. Full access to the data was obtained from www.dhsprogram.com, in compliance with the data sharing policy. The survey authority obtained ethical approval from the Government of Bangladesh, and informed consent was secured from all respondents before they participated in the interviews.

## 3 Results

### 3.1 Basic Characteristics of the Study Population

A total of 3950 children under five were included in the study, with nearly half (49%) being girls. The majority of the children (85%) had average or larger birth sizes. Educational attainment was relatively high, with 54% of mothers having completed secondary education. Approximately 60% of mothers had fewer than four antenatal care visits. Around one‐third of the mothers were short in stature, while 13% were underweight. The majority of participants (74%) resided in rural areas (Table 1).

**TABLE 1:** Background characteristics of the study population.

Variables	*n* (%)
Child sex	
Male	2026 (51.1)
Female	1924 (48.9)
Child age (months)	
0–11	879 (22.4)
12–23	756 (20.0)
24–35	769 (19.6)
36–47	766 (18.9)
48–59	780 (19.1)
Birth order of child	
1st	1480 (37.5)
2nd	1382 (34.6)
3rd	692 (18.0)
≥ 4	396 (9.9)
Birth size of child (*n* = 2398)	
Average or larger	2063 (85.3)
Smaller than average	335 (14.7)
Mother's age (years)	
15–19	354 (9.7)
20–24	1234 (31.6)
25–34	1921 (47.6)
35 and above	441 (11.1)
Maternal occupation	
Currently working	956 (25.6)
Currently not working	2994 (74.5)
Maternal attitude toward wife‐beating	
Not justified	3477 (87.0)
Justified	473 (13.0)
Maternal decision‐making autonomy	
Practiced	3273 (83.8)
Not practiced	677 (16.2)
Maternal education	
No education	240 (6.0)
Primary	894 (21.6)
Secondary	2062 (54.4)
Higher	754 (18.0)
ANC visit (*n* = 2298)	
< 4	1352 (60.0)
≥ 4	946 (40.0)
Maternal stature	
Short (< 150 cm)	1440 (36.6)
Tall (≥ 150 cm)	2510 (63.4)
Maternal underweight (BMI < 18.5)	
No	3432 (87.3)
Yes	518 (12.7)
Father's education	
No education	607 (15.2)
Primary	1198 (30.6)
Secondary	1371 (35.6)
Higher	774 (18.7)
Exposed with media	
TV/Radio/Newspaper	1839 (46.7)
Not at all	2111 (53.3)
Toilet facility	
Improved	3240 (82.7)
Unimproved	710 (17.3)
Wealth index	
Poorest	841 (20.5)
Poorer	773 (20.2)
Middle	789 (20.6)
Richer	773 (19.7)
Richest	774 (19.0)
Place of residence	
Urban	1264 (25.9)
Rural	2686 (74.1)

### 3.2 Prevalence of Undernutrition Among Children

The prevalence of different forms of undernutrition among children under 5 years old is presented in Table 2. According to the CIAF, the overall prevalence of undernutrition was 33.7%. Among these, 18.9% experienced MFU: 4.7% were both wasted and underweight, 10.8% were both stunted and underweight, and 3.4% experienced wasting, stunting, and underweight simultaneously. Meanwhile, 14.8% had SFU, with 3.0% being only wasted, 8.8% only stunted, and 3.0% only underweight. In comparison, when using conventional anthropometric indices, the prevalence of stunting, wasting, and underweight was 23.0%, 11.1%, and 21.9%, respectively.

**TABLE 2:** Prevalence of different forms of undernutrition among under 5 children.

Undernutrition indicators	Description		Percentage
CIAF	SFU	Wasting only	3.0
		Stunting only	8.8
		Underweight only	3.0
	MFU	Wasting and underweight	4.7
		Stunting and underweight	10.8
		Wasting, stunting, and underweight	3.4
Conventional Anthropometric Index	Stunting		23.0
	Wasting		11.1
	Underweight		21.9

Abbreviations: CIAF, composite index of anthropometric failure; MFU, multiple form of undernutrition; SFU, single form of undernutrition.

Younger children (0–11 months) and those of lower birth order had significantly lower MFU (*p* < 0.001) and CIAF (*p* < 0.001). Children with smaller than average birth sizes had a significantly higher prevalence of SFU (*p* = 0.005), MFU (*p* < 0.001), and CIAF (*p* < 0.001). Those born to older mothers (≥ 35 years) had a higher prevalence of MFU (*p* < 0.001) and CIAF (*p* = 0.03). Additionally, maternal approval of wife‐beating was linked to a higher prevalence of MFU (*p* = 0.015). Parental education also showed a strong influence, with children of uneducated parents having a significantly higher prevalence of MFU (*p* < 0.001) and CIAF (*p* < 0.001). Limited ANC visits (< 4) were associated with a higher prevalence of MFU (*p* < 0.001) and CIAF (*p* = 0.001). Children of mothers with short stature (< 150 cm) had a higher prevalence of SFU (*p* < 0.001), MFU (*p* < 0.001), and CIAF (*p* < 0.001), and maternal underweight was similarly linked to a higher prevalence of MFU (*p* < 0.001) and CIAF (*p* < 0.001). At the household level, children from families with unimproved toilet facilities and those in the poorest wealth quintile had a significantly higher prevalence of MFU (*p* < 0.001) and CIAF (*p* < 0.001). Lack of maternal media exposure was also associated with a higher prevalence of MFU (*p* < 0.001) and CIAF (*p* = 0.002). Regional disparities were evident, with children residing in rural areas showing a higher prevalence of MFU (*p* = 0.028). Furthermore, the highest prevalence of MFU (*p* < 0.001) and CIAF (*p* < 0.001) was observed among children living in the Sylhet division (Table 3). Additionally, variations in specific forms of undernutrition under SFU and MFU across different background characteristics are presented in Table S4.

**TABLE 3:** Prevalence of child undernutrition across the background characteristics among under‐5 children in Bangladesh.

	SFU	*p*	MFU	*p*	CIAF	*p*
Child sex						
Male	15.0 [13.3, 16.8]	0.795	18.5 [16.6, 20.6]	0.589	33.5 [31.2, 35.8]	0.808
Female	14.6 [12.9, 16.5]		19.3 [17.3, 21.4]		33.9 [31.3, 36.6]	
Child age (months)						
0–11	13.7 [11.3, 16.4]	0.078	11.7 [9.5, 14.3]	< 0.001	25.3 [22.0, 29.0]	< 0.001
12–23	17.2 [14.2, 20.6]		18.0 [15.2, 21.2]		35.2 [31.5, 39.0]	
24–35	16.1 [13.4, 19.2]		22.1 [18.9, 25.5]		38.2 [34.3, 42.2]	
36–47	15.1 [12.6, 18.1]		21.1 [17.9, 24.6]		36.2 [32.5, 40.0]	
48–59	11.9 [9.7, 14.6]		22.9 [19.7, 26.4]		34.8 [31.1, 38.8]	
Birth order of child						
1st	14.5 [12.4, 16.8]	0.481	16.2 [14.2, 18.5]	< 0.001	30.7 [27.9, 33.6]	< 0.001
2nd	14.1 [12.2, 16.3]		18.6 [16.4, 21.0]		32.7 [30.0, 35.5]	
3rd	15.2 [12.7, 18.3]		19.6 [16.5, 23.0]		34.8 [30.9, 38.9]	
≥ 4	17.6 [13.7, 22.2]		28.7 [23.5, 34.6]		46.3 [41.1, 51.5]	
Birth size of child (*n* = 2398)						
Average or larger	14.4 [12.8, 16.2]	0.005	15.7 [14.0, 17.7]	< 0.001	30.2 [27.8, 32.6]	< 0.001
Smaller than average	21.8 [16.8, 27.9]		24.7 [19.6, 30.5]		46.5 [39.9, 53.2]	
Mother's age (years)						
15–19	16.0 [12.1, 20.9]	0.175	12.7 [9.5, 16.8]	< 0.001	28.7 [23.8, 34.3]	0.030
20–24	12.8 [10.8, 15.1]		20.6 [17.9, 23.7]		33.4 [30.3, 36.8]	
25–34	16.0 [14.2, 17.9]		17.5 [15.6, 19.5]		33.4 [31.1, 35.9]	
35 and above	14.4 [11.1, 18.4]		25.3 [20.6, 30.8]		39.7 [34.6, 45.1]	
Maternal occupation						
Currently working	15.1 [12.9, 17.6]	0.770	18.9 [16.1, 22.1]	0.968	34.0 [30.6, 37.6]	0.824
Currently not working	14.7 [13.3, 16.2]		18.9 [17.1, 20.7]		33.6 [31.4, 35.8]	
Maternal attitude toward wife‐beating						
Justified	14.2 [11.2, 17.8]	0.702	23.7 [19.4, 28.6]	0.015	37.9 [32.7, 43.3]	0.082
Not justified	14.9 [13.6, 16.3]		18.2 [16.6, 19.8]		33.1 [31.1, 35.0]	
Maternal decision‐making autonomy						
Practiced	14.7 [13.4, 16.1]	0.807	18.4 [16.9, 20.1]	0.153	33.2 [31.2, 35.2]	0.152
Not practiced	15.1 [12.4, 18.3]		21.2 [17.6, 25.2]		36.3 [32.2, 40.6]	
Maternal education						
No education	14.0 [10.2, 18.9]	0.861	40.1 [33.1, 47.6]	< 0.001	54.2 [47.4, 60.8]	< 0.001
Primary	15.2 [13.0, 17.8]		23.6 [20.6, 26.9]		38.8 [35.4, 42.4]	
Secondary	15.0 [13.4, 16.8]		17.5 [15.6, 19.5]		32.5 [30.1, 35.0]	
Higher	13.8 [11.0, 17.3]		10.4 [8.0, 13.3]		24.2 [20.7, 28.2]	
ANC visit (*n* = 2298)						
< 4	14.8 [12.7, 17.1]	0.210	20.6 [18.1, 23.3]	< 0.001	35.3 [32.3, 38.4]	0.001
≥ 4	17.2 [14.3, 20.5]		10.5 [8.6, 12.7]		27.7 [24.4, 31.1]	
Maternal stature						
Short (< 150 cm)	18.5 [16.4, 20.9]	< 0.001	26.1 [23.6, 28.7]	< 0.001	44.6 [41.7, 47.6]	< 0.001
Tall (≥ 150 cm)	12.6 [11.2, 14.2]		14.7 [13.1, 16.6]		27.4 [25.2, 29.6]	
Maternal underweight (BMI < 18.5)						
No	14.5 [13.3, 15.9]	0.340	17.5 [16.0, 19.1]	< 0.001	32.1 [30.1, 34.1]	< 0.001
Yes	16.5 [13.0, 20.7]		28.2 [24.0, 32.8]		44.7 [39.9, 49.6]	
Father's education						
No education	16.8 [13.9, 20.1]	0.201	27.2 [23.3, 31.5]	< 0.001	44.0 [39.2, 48.8]	< 0.001
Primary	14.6 [12.6, 16.8]		21.4 [18.8, 24.3]		36.0 [32.7, 39.3]	
Secondary	13.4 [11.5, 15.6]		17.7 [15.5, 20.1]		31.1 [28.4, 33.8]	
Higher	16.2 [13.5, 19.3]		10.3 [8.1, 13.0]		26.5 [23.2, 30.2]	
Exposed with media						
Yes	15.3 [13.5, 17.3]	0.504	15.6 [13.8, 17.6]	< 0.001	30.9 [28.6, 33.3]	0.002
Not at all	14.4 [12.8, 16.1]		21.7 [19.7, 23.9]		36.1 [33.6, 38.7]	
Toilet facility						
Improved	14.5 [13.2, 16.0]	0.340	17.4 [15.9, 19.1]	< 0.001	32.0 [30.0, 34.0]	< 0.001
Unimproved	16.1 [13.4, 19.2]		25.8 [22.3, 29.6]		41.9 [37.8, 46.1]	
Wealth index						
Poorest	16.2 [13.8, 18.9]	0.543	28.9 [25.7, 32.4]	< 0.001	45.1 [41.3, 49.0]	< 0.001
Poorer	15.5 [12.8, 18.6]		22.4 [19.2, 25.8]		37.8 [33.8, 42.1]	
Middle	13.0 [10.8, 15.5]		18.4 [15.6, 21.6]		31.4 [28.1, 34.9]	
Richer	14.2 [11.7, 17.2]		12.5 [10.2, 15.3]		26.7 [23.3, 30.5]	
Richest	15.2 [12.0, 19.0]		11.4 [8.8, 14.7]		26.6 [22.7, 30.9]	
Place of residence						
Urban	16.2 [13.5, 19.3]	0.235	16.1 [13.6, 18.9]	0.028	32.3 [28.7, 36.1]	0.389
Rural	14.3 [13.0, 15.7]		19.9 [18.1, 21.8]		34.2 [32.0, 36.4]	
Division						
Barisal	15.0 [11.7, 19.2]	0.576	22.2 [17.5, 27.7]	0.001	29.9 [25.6, 34.6]	0.001
Chattogram	14.0 [11.6, 16.7]		20.1 [16.3, 24.5]		37.2 [32.2, 42.6]	
Dhaka	15.4 [12.3, 19.1]		14.5 [11.4, 18.3]		34.1 [29.2, 39.3]	
Khulna	12.7 [9.9, 16.0]		16.0 [12.8, 19.9]		28.7 [24.5, 33.2]	
Mymensingh	17.5 [14.7, 20.7]		22.4 [19.4, 25.7]		39.9 [35.8, 44.1]	
Rajshahi	13.3 [9.9, 17.6]		17.3 [13.7, 21.6]		30.6 [26.1, 35.6]	
Rangpur	15.0 [12.0, 18.5]		19.9 [16.3, 24.0]		34.8 [30.5, 39.4]	
Sylhet	16.7 [13.4, 20.7]		26.8 [22.3, 31.8]		43.5 [39.3, 47.8]	

Abbreviations: CIAF, composite index of anthropometric failure; MFU, multiple form of undernutrition; SFU, single form of undernutrition.

### 3.3 Factors Associated With Undernutrition Among Children

The factors associated with CIAF are presented in Table 4. Child age was a significant determinant, with older children at a higher risk of experiencing CIAF compared to the youngest aged 0–11 months. Children with a higher birth order (≥ 4) had a significantly increased risk of experiencing CIAF (ARR: 1.30, 95% CI: 1.08–1.58, *p* = 0.007) compared to firstborns. Similarly, children born smaller than average were at a 45% higher risk of experiencing CIAF (ARR: 1.45, 95% CI: 1.24–1.70, *p* < 0.001) than those born with an average or larger size. Children of uneducated mothers had a significantly higher risk of CIAF (ARR: 1.49, 95% CI: 1.16–1.93, *p* = 0.002). Maternal short stature (< 150 cm) further increased the risk, with children of shorter mothers 54% more likely to experience CIAF (ARR: 1.54, 95% CI: 1.40–1.70, *p* < 0.001). Similarly, maternal underweight was associated with a greater risk of CIAF (ARR: 1.28, 95% CI: 1.13–1.46, *p* < 0.001). Children from the poorest households were significantly more likely to experience CIAF (ARR: 1.25, 95% CI: 1.02–1.53, *p* = 0.034). Regional disparities were evident, with children in Sylhet having a significantly higher risk of CIAF (ARR: 1.22, 95% CI: 1.02–1.47, *p* = 0.029) compared to those in Dhaka.

**TABLE 4:** Factors associated with CIAF among under‐five children in Bangladesh, 2022.

Variables	CIAF			
	Unadjusted RR (95% CI)	*p*	Adjusted RR (95% CI)^a^	*p*
Child age (months)				
0–11	Ref.		Ref.	
12–23	1.39 (1.18, 1.63)	< 0.001	1.36 (1.15, 1.61)	< 0.001
24–35	1.51 (1.27, 1.79)	< 0.001	1.49 (1.26, 1.76)	< 0.001
36–47	1.43 (1.20, 1.69)	< 0.001	1.42 (1.19, 1.68)	< 0.001
48–59	1.37 (1.16, 1.62)	< 0.001	1.34 (1.13, 1.60)	0.001
Birth order of child				
1st	Ref.		Ref.	
2nd	1.07 (0.95, 1.20)	0.277	1.08 (0.94, 1.25)	0.251
3rd	1.13 (0.98, 1.31)	0.084	1.12 (0.93, 1.34)	0.239
≥ 4	1.51 (1.31, 1.73)	< 0.001	1.30 (1.08, 1.58)	0.007
Birth size of child (*n* = 2398)				
Average or larger	Ref.		Ref.	
Smaller than average	1.54 (1.31, 1.81)	< 0.001	1.45 (1.24, 1.70)	< 0.001
Mother's age (years)				
15–19	0.72 (0.58, 0.9)	0.003	1.01 (0.78, 1.3)	0.968
20–24	0.84 (0.71, 0.99)	0.037	1.07 (0.88, 1.32)	0.489
25–34	0.84 (0.73, 0.97)	0.021	1.02 (0.87, 1.18)	0.838
35 and above	Ref.		Ref.	
Maternal attitude toward wife‐beating				
Not justified	Ref.		Ref.	
Justified	1.15 (0.99, 1.33)	0.074	1.08 (0.93, 1.24)	0.317
Maternal decision‐making autonomy				
Not practiced	Ref.		Ref.	
Practiced	0.91 (0.81, 1.03)	0.146	0.92 (0.82, 1.03)	0.146
Maternal education				
No education	2.24 (1.82, 2.74)	< 0.001	1.49 (1.16, 1.93)	0.002
Primary	1.60 (1.35, 1.90)	< 0.001	1.18 (0.94, 1.47)	0.151
Secondary	1.34 (1.13, 1.59)	0.001	1.15 (0.95, 1.41)	0.159
Higher	Ref.		Ref.	
ANC visit (*n* = 2298)				
≥ 4 times	Ref.		Ref.	
< 4 times	1.28 (1.10, 1.48)	0.001	1.11 (0.96, 1.30)	0.166
Maternal stature				
Tall (≥ 150 cm)	Ref.		Ref.	
Short (< 150 cm)	1.63 (1.48, 1.80)	< 0.001	1.54 (1.40, 1.70)	< 0.001
Maternal underweight				
No	Ref.		Ref.	
Yes	1.39 (1.23, 1.58)	< 0.001	1.28 (1.13, 1.46)	< 0.001
Father's education				
No education	1.66 (1.39, 1.98)	< 0.001	1.03 (0.83, 1.27)	0.814
Primary	1.36 (1.16, 1.59)	< 0.001	0.94 (0.77, 1.14)	0.531
Secondary	1.17 (1.00, 1.37)	0.046	0.95 (0.80, 1.14)	0.601
Higher	Ref.		Ref.	
Exposed with media				
TV/Radio/Newspaper	Ref.		Ref.	
Not at all	1.17 (1.06, 1.29)	0.002	0.99 (0.89, 1.11)	0.907
Toilet facility				
Improved	Ref.		Ref.	
Unimproved	1.31 (1.17, 1.47)	< 0.001	1.00 (0.88, 1.13)	0.991
Wealth index				
Poorest	1.70 (1.42, 2.02)	< 0.001	1.25 (1.02, 1.53)	0.034
Poorer	1.42 (1.18, 1.72)	< 0.001	1.19 (0.97, 1.45)	0.090
Middle	1.18 (0.97, 1.43)	0.090	1.07 (0.88, 1.30)	0.502
Richer	1.01 (0.82, 1.23)	0.959	0.97 (0.79, 1.19)	0.778
Richest	Ref.		Ref.	
Division				
Dhaka	Ref.		Ref.	
Barisal	1.25 (1.01, 1.53)	0.037	1.15 (0.93, 1.42)	0.186
Chattogram	1.14 (0.92, 1.41)	0.228	1.04 (0.85, 1.28)	0.697
Khulna	0.96 (0.77, 1.19)	0.698	0.95 (0.78, 1.16)	0.618
Mymensingh	1.33 (1.11, 1.6)	0.002	1.10 (0.92, 1.31)	0.312
Rajshahi	1.02 (0.82, 1.27)	0.829	0.97 (0.78, 1.20)	0.789
Rangpur	1.16 (0.96, 1.42)	0.130	1.05 (0.87, 1.27)	0.582
Sylhet	1.45 (1.21, 1.74)	< 0.001	1.22 (1.02, 1.47)	0.029

Abbreviations: CIAF, composite index of anthropometric failure; CI, confidence interval; Ref, reference; RR, risk ratio.

^a^ All values reported were adjusted for variables with a *p*‐value < 0.25 in the unadjusted model, except for ANC visits and birth size. For these two variables, adjustments were made separately for all other variables with a *p*‐value < 0.25 in the unadjusted model, due to a large number of missing values.

## 4 Discussion

This study used data from the 2022 BDHS to estimate the prevalence and determinants of CIAF among under‐five children in Bangladesh. The findings revealed that around one‐third of children experienced some form of anthropometric failure. The analysis identified several associated factors of CIAF, including child‐specific characteristics such as age, birth order, and birth size; maternal factors such as education, underweight, and stature; household wealth status; and region.

The high prevalence of various forms of child undernutrition observed in this study reflected the ongoing challenges faced in South Asia and other LMICs (UNICEF, WHO, and World Bank 2023). Over the years, child undernutrition in Bangladesh has shown a gradual decline, as reflected in CIAF rates, which dropped from 48.3% in 2014 to 38% in 2017 and further to 34% in this study (Chowdhury, Khan, Rashid, et al. 2021). Similarly, while MFU remained constant at 19% between 2017 and 2022, SFU declined from 19% to 14.8% (Chowdhury, Khan, Rashid, et al. 2021). Despite these improvements, recent trends suggested a worsening of acute undernutrition in Bangladesh (NIPORT & ICF 2024). Both stunting and underweight decreased (13.5%–10.8%), while both wasting and underweight increased (3.1%–4.7%) between 2017 and 2022 (Chowdhury, Khan, Rashid, et al. 2021). Additionally, while only stunting declined (14.3%–8.8%), only wasting (2.1%–3.0%) and only underweight (2.4%–3.0%) showed an increasing trend (Chowdhury, Khan, Rashid, et al. 2021). Several factors might have contributed to the rising trend of acute undernutrition among infants and young children. First, rising inflation has significantly impacted food security and nutrition. Inflation in Bangladesh had increased from 5.8% in 2016/17 to 9% in 2022/23, with food price inflation rising from 6% to 8.7% (BBS 2024). A 5% rise in food prices elevated wasting risk by 9%, forcing households to reduce the quality and diversity of their diets, as well as their access to healthcare services (Headey and Ruel 2023). Second, exposure to environmental pathogens, poor water, sanitation, and hygiene conditions could lead to environmental enteropathy, which might contribute to growth faltering and wasting (Global Hunger Index 2018). Bangladesh has experienced significant environmental degradation over the years in terms of air pollution, water pollution, deforestation, soil degradation, biodiversity loss, and climate change (Lima et al. 2024).

Older children were found to be more at risk of undernourishment, a finding consistent with earlier studies (Bornee et al. 2025; Chowdhury, Khan, and Mondal 2021; Chowdhury, Khan, Rashid, et al. 2021; Kundu et al. 2022). As children grow, their nutritional needs increase, yet in Bangladesh, their diets are often characterized by low diversity, cereal‐based, and poor nutrient content (Arsenault et al. 2013; Sanin et al. 2018). Such inadequate feeding practices fail to provide the vitamins and minerals needed post‐breastfeeding (Sumon et al. 2023). Furthermore, older children may also face reduced attention and fewer resources in large households, contributing to their higher risk of undernourishment (Chowdhury, Khan, Rashid, et al. 2021).

Similar to other studies, higher birth order was associated with undernutrition (Anik et al. 2021; Bornee et al. 2025; Chowdhury, Khan, and Mondal 2021; Chowdhury, Khan, Rashid, et al. 2021). Children with higher birth order were more likely to be unintended, which could result in reduced parental care, including fewer antenatal and postnatal checkups (Kamal and Islam 2011). Furthermore, as family size increased, household resources such as food and caregiving became more limited, resulting in inadequate dietary intake and poorer nutritional outcomes among children (Howell et al. 2016; Kundu et al. 2022). Likewise, our findings indicate that birth size has consistently been identified as a risk factor for undernutrition (Fenta et al. 2021; Siddiqa et al. 2024). Smaller birth size often reflects poor maternal nutrition and inadequate prenatal care, both of which can hinder fetal growth and contribute to low birth weight (LBW) (Marshall et al. 2022). Infants with LBW are predisposed to developmental delays, increasing their susceptibility to early‐life undernutrition (Rahman et al. 2016; Upadhyay et al. 2019).

Maternal education has been shown to significantly reduce the likelihood of child undernutrition, a finding well‐documented in Bangladesh (Anik et al. 2021; Bornee et al. 2025; Chowdhury, Khan, and Mondal 2021; Chowdhury, Khan, et al. 2022; Chowdhury, Rahman, et al. 2022; Kundu et al. 2022; Sumon et al. 2023) and other countries (Fenta et al. 2021; Siddiqa et al. 2024; Vollmer et al. 2017). Educated mothers possess greater knowledge, autonomy, resource control, and employment opportunities, which positively impact child feeding, healthcare practices, and overall child health and nutrition (Mensch et al. 2019; Sarkar et al. 2023; Vollmer et al. 2017).

Similar to earlier studies, it was evident that maternal undernourishment had an association with child undernutrition (Bornee et al. 2025; Chowdhury, Khan, and Mondal 2021; Chowdhury, Khan, et al. 2022; Chowdhury, Rahman, et al. 2022; Kundu et al. 2022; Sumon et al. 2023). Maternal undernutrition as well as short stature are associated with intrauterine growth retardation and LBW at delivery, both of which increase the risk of child undernutrition (Black et al. 2013; Khaliq et al. 2024; Young et al. 2015).

Our findings align with previous studies indicating that child undernutrition was more reflected among the poorest households (Anik et al. 2021; Bornee et al. 2025; Chowdhury, Khan, et al. 2022; Islam and Biswas 2020; Kundu et al. 2022; Sumon et al. 2023). It is a global phenomenon, and such trends are observed in numerous settings, particularly in LMICs (Siddiqa et al. 2024; Vollmer et al. 2017). Families with higher socioeconomic status benefit from better access to nutritious food, quality healthcare, and better living conditions, all of which contribute to healthier growth and reduced risks of undernutrition in children (Rahman et al. 2021). By contrast, children living in poor households are often exposed to food insecurity, inadequate healthcare, and unhealthy living environments, creating a compounding effect that perpetuates the cycle of undernutrition.

Our study identified significant regional disparities in child undernutrition, with children from the Sylhet division being the most affected, which aligns with earlier studies (Bornee et al. 2025; Islam and Biswas 2020). Sylhet lags behind other regions in critical determinants of child nutrition. Sylhet has the lowest female literacy rates, poor school attendance, high gender inequality, and the lowest levels of women's empowerment and ANC visits (Das et al. 2022; Sanin et al. 2023). Furthermore, the region's unique geographical features, including its wetland areas and tea estates, exacerbate these challenges (Sanin et al. 2023). These locations are home to large populations of marginalized communities, which further exacerbate the risk.

The persistent prevalence of child undernutrition in Bangladesh highlights the urgent need for more effective and targeted nutrition‐specific interventions along with nutrition‐sensitive interventions despite past efforts taken under the National Nutrition Policy (2015) and the Second National Plan of Action for Nutrition (2016–2025). Key policy recommendations include improving parental education on nutrition and child feeding practices, as well as implementing targeted, community‐based support programs for vulnerable populations. These programs should encompass financial assistance, food subsidies, and supplementary feeding for LBW children and undernourished pregnant and lactating women. Additionally, addressing the economic impact of rising food prices through social safety nets is crucial. To tackle regional disparities, especially in areas like Sylhet, policies should prioritize improving access to healthcare, education, and nutritional resources in marginalized regions, with specific attention to the unique challenges faced by rural, Haor, and tea estate populations. However, the effectiveness of current policies is hindered by institutional bottlenecks, such as overlapping mandates, poor coordination, and limited accountability (Saha et al. 2015). To overcome these constraints, policies should focus on strengthening inter‐ministerial collaboration, enhancing local‐level engagement, ensuring data‐driven decision‐making, and establishing robust monitoring and evaluation frameworks. Integrating these strategies into existing frameworks would help create a more cohesive, efficient approach to reducing child undernutrition and achieving sustainable development goals.

This study has several strengths and limitations. A key strength is its use of the most recent and nationally representative dataset, allowing for a broad and current understanding of undernutrition among under‐five children in Bangladesh. By focusing on different forms of undernutrition, this study offers unique insights into nutritional challenges. The application of sampling weights during analysis ensures that the findings are generalizable at the national level. However, the cross‐sectional nature of the data limits causal inference. The unavailability of some important variables like child dietary intake, food insecurity, and parental behavior further limits the study findings.

## 5 Conclusion

Child undernutrition is a significant public health concern in Bangladesh, influenced by factors such as older child age, higher birth order, smaller birth size, maternal undernutrition, lack of maternal education, household poverty, and regional disparities, particularly in the Sylhet division. The study underscores the urgent need for strengthened, nutrition‐specific, community‐based interventions targeting mothers and children. In addition, efforts to expand family planning services, improve access to healthcare, enhance food assistance programs, and increase household income are essential to reducing undernutrition in Bangladesh.

## Author Contributions

**Rafid Hassan:** conceptualization (lead), data curation (lead), formal analysis (lead), investigation (lead), methodology (lead), resources (lead), software (lead), supervision (equal), validation (equal), visualization (lead), writing – original draft (equal), writing – review and editing (equal). **Shahadoth Hossain:** writing – original draft (equal), writing – review and editing (equal). **Md Mahbub Alam:** writing – original draft (equal), writing – review and editing (equal). **Sanjib Saha:** supervision (equal), validation (equal), writing – original draft (equal), writing – review and editing (equal). **Tanjina Akter:** writing – original draft (equal), writing – review and editing (equal). **Masum Ali:** writing – review and editing (equal). **Md Ruhul Amin:** supervision (equal), writing – original draft (equal), writing – review and editing (equal).

## Supporting information


**Table S1.** Classification of composite index of anthropometric failure among under‐five children.
**Table S2**. Variable definitions.
**Table S3**. Variance Inflation Factor (VIF) of the variables.
**Table S4**. Prevalence of different forms of undernutrition across background characteristics.

## Data Availability

All data are freely available from the DHS: https://www.dhsprogram.com/Countries/Country‐Main.cfm?ctry_id=1&c=Bangladesh&Country=Bangladesh&cn=&r=4.
