# Supplementary material for: Prevalence and Determinants of the Composite Index of Anthropometric Failure in Children Under Five Years in Bangladesh: Insights From a National Survey
Source: Food Sci Nutr. 2025 Jun 22;13(6):e70499. doi: 10.1002/fsn3.70499 (PMC12183109; doi:10.1002/fsn3.70499)
Supplement: Supplementary file 1 — Table S1. Classification of composite index of anthropometric failure among under‐five children. Table S2. Variable definitions. Table S3. Variance Inflation Factor (VIF) of the variables. Table S4. Prevalence of different forms of undernutrition across background characteristics. [file FSN3-13-e70499-s001.docx]

**Supplementary Table 1.** Classification of composite index of anthropometric failure among under-five children

| **Group** | **Description** | **Wasting** | **Stunting** | **Underweight** |
| --- | --- | --- | --- | --- |
| A | No failure | No | No | No |
| B | Wasting only | Yes | No | No |
| C | Wasting and Underweight | Yes | No | Yes |
| D | Wasting, Stunting, and Underweight | Yes | Yes | Yes |
| E | Stunting and Underweight | No | Yes | Yes |
| F | Stunting only | No | Yes | No |
| Y | Underweight only | No | No | Yes |

***Note:*** No anthropometric failure (NAF) = A; Anthropometric failure (AF) = Σ of B, C, D, E, F, Y; Multiple form of undernutrition= Σ of C, D, E; Single form of undernutrition = Σ of B, F, Y.

**Supplementary Table 2.** Variable definitions

| **Variables** | **Description** | **Measurement** |
| --- | --- | --- |
| Child sex | Sex differential of children | Male, Female |
| Child age (in months) | Age of the children at the time of data collection | 0–11, 12–23, 24–35, 36–47, 48–59 |
| Birth order of child | Birth order is the chronological order of sibling births in a family | One, two, three, four and above |
| Birth size of child | Size of the baby after birth | Average or larger, Smaller than average |
| Mother’s age | Age of mothers at the time of data collection | 15–19, 20–24,  25–34,  35 and above |
| Maternal occupation | Mothers engaged in economic activity at the time of data collection | Currently working, Currently not working |
| Maternal attitude towards wife beating | Mother's beliefs, opinions, or stance  on the acceptability or justification of physical violence by a husband against his wife | Justified, Not justified |
| Maternal decision making autonomy | In the BDHS surveys, a woman’s decision-making power is assessed on the following three themes: 1) a woman who usually decides on her healthcare  2) a woman who usually decides on large household purchases and 3) a woman who usually decides on visits to family or relatives. | Practiced, Not practiced |
| Maternal education | Educational status of mother | No education, Primary, Secondary,  Higher |
| ANC visit | Medical surveillance and review performed during pregnancy for the early detection of possible complications of pregnancy | <4 times,  ≥4 times |
| Maternal stature | Height of mother in cm | Short (< 150 cm),  Tall (≥ 150 cm) |
| Maternal underweight | Body Mass Index of mother<18.5 | Yes, No |
| Father’s education | Educational status of father | No education, Primary, Secondary,  Higher |
| Exposed with media | Media exposure through television, radio and newspaper /magazine has been defined as exposure to at least one media that exposes to at least once a week | Yes, Not at all |
| Toilet facility | **Improved:** flush - to piped sewer system, flush - to septic tank, flush - to pit latrine, flush - don't know where, pit latrine – ventilated improved pit, pit latrine - with slab, composting toilet; **Unimproved:** flush - to somewhere else, pit latrine - without slab / open pit, bucket toilet, hanging toilet/latrine, other | Improved, Unimproved |
| Wealth index | Wealth index in the DHS surveys is calculated, by the DHS authority, based on information on household characteristics and assets using principal component analysis. Then households are classified into quintiles based on the values of the wealth index, where households with  lower values of the index is considered as poorest and vice-versa | Poorest, poorer, middle, richer, richest |
| Place of residence | The geographic location or address where an individual lives as either an urban area (city or town) or a rural area (countryside or less densely populated region). | Urban, Rural |
| Division | There are eight administrative divisions in Bangladesh: Barishal, Chattogram, Dhaka, Khulna, Mymensingh, Rajshahi, Rangpur, and Sylhet. | Barishal, Chattogram, Dhaka, Khulna  Mymensingh,  Rajshahi, Rangpur, Sylhet |

**Supplementary Table 3.** Variance Inflation Factor (VIF) of the variables

| **Variables** | **VIF** |
| --- | --- |
|  | **CIAF** |
| Child age (months) | 1.04 |
| Birth order of child | 1.9 |
| Birth size of child | 1.02 |
| Mother's age (years) | 1.76 |
| Maternal attitude toward wife-beating | 1.01 |
| Maternal decision-making autonomy | 1.04 |
| Maternal education | 1.66 |
| ANC visit | 1.17 |
| Maternal stature | 1.06 |
| Maternal underweight | 1.07 |
| Father's education | 1.70 |
| Exposed with media | 1.15 |
| Toilet facility | 1.20 |
| Wealth index | 1.71 |
| Division | 1.03 |

**Supplementary Table 4.** Prevalence of different forms of undernutrition across background characteristics

| **Variable** | **Wasting only** | **Wasting + Underweight** | **Stunting + Wasting + Underweight** | **Stunting + Underweight** | **Stunting only** | **Underweight only** |
| --- | --- | --- | --- | --- | --- | --- |
| **Child sex** |  |  |  |  |  |  |
| Male | 3.0 [2.3, 3.9] | 4.2 [3.4, 5.3] | 3.5 [2.7, 4.6] | 10.8 [9.3, 12.4] | 9 [7.7, 10.5] | 3 [2.3, 3.9] |
| Female | 3.0 [2.2, 4.0] | 5.3 [4.3, 6.5] | 3.2 [2.4, 4.3] | 10.8 [9.3, 12.4] | 8.7 [7.2, 10.4] | 3 [2.2, 3.9] |
| **Child age (months)** |  |  |  |  |  |  |
| 0–11 | 3.9 [2.8, 5.6] | 4.9 [3.5, 6.8] | 1.2 [0.7, 2.2]** | 5.6 [4.1, 7.5]*** | 7.2 [5.5, 9.4]*** | 2.5 [1.5, 4.2] |
| 12–23 | 1.6 [0.9, 3.0] | 4.2 [2.9, 6.0] | 3.3 [2.2, 5.0] | 10.5 [8.4, 13.0] | 13.6 [10.9, 16.8] | 2.0 [1.1, 3.5] |
| 24–35 | 3.4 [2.1, 5.3] | 4.8 [3.4, 6.7] | 3.3 [2.2, 5.1] | 13.9 [11.5, 16.8] | 10.3 [8.1, 13.0] | 2.4 [1.5, 3.9] |
| 36–47 | 3.0 [1.9, 4.5] | 4.8 [3.3, 6.8] | 4.9 [3.4, 7.1] | 11.3 [9.0, 14.2] | 8.2 [6.3, 10.7] | 4.0 [2.6, 6.0] |
| 48–59 | 2.9 [1.9, 4.5] | 5.1 [3.7, 6.9] | 4.5 [3.1, 6.4] | 13.3 [10.9, 16.2] | 4.8 [3.4, 6.8] | 4.2 [2.9, 6.1] |
| **Birth order of child** |  |  |  |  |  |  |
| 1st | 3.0 [2.2, 4.2] | 4 [3.1, 5.2] | 2.7 [2.0, 3.8]** | 9.5 [7.9, 11.3]** | 8.5 [6.9, 10.4] | 3.0 [2.1, 4.3] |
| 2nd | 2.8 [2.0, 4.0] | 5.1 [3.9, 6.6] | 3.2 [2.3, 4.4] | 10.3 [8.7, 12.1] | 8.3 [6.9, 10.0] | 3.0 [2.1, 4.2] |
| 3rd | 3.3 [2.2, 5.2] | 5.2 [3.7, 7.4] | 3.0 [1.9, 4.6] | 11.4 [9.0, 14.2] | 9.8 [7.6, 12.5] | 2.1 [1.2, 3.7] |
| ≥4 | 2.7 [1.1, 6.2] | 5.4 [3.4, 8.2] | 7.2 [4.1, 12.3] | 16.1 [12.8, 20.2] | 10.3 [7.6, 14.0] | 4.6 [2.8, 7.4] |
| **Birth size of child (n=2,398)** |  |  |  |  |  |  |
| Average or larger | 2.8 [2.2, 3.7] | 4.2 [3.4, 5.2] | 2.5 [1.8, 3.3] | 9.1 [7.8, 10.5]** | 9.4 [8.1, 11.0]** | 2.2 [1.6, 3.0] |
| Smaller than average | 4.1 [2.0, 8.3] | 7.3 [4.3, 12.2] | 3.3 [1.8, 6.0] | 14.1 [10.5, 18.7] | 14.8 [10.9, 19.7] | 2.9 [1.5, 5.8] |
| **Mother's age (years)** |  |  |  |  |  |  |
| 15–19 | 2.1 [1.0, 4.3] | 4.7 [2.8, 7.8] | 1.7 [0.8, 3.5]** | 6.4 [4.1, 9.6]** | 11.2 [8.0, 15.3] | 2.7 [1.4, 5.3] |
| 20–24 | 2.6 [1.8, 3.9] | 5.0 [3.7, 6.7] | 2.8 [1.9, 4.0] | 12.9 [10.8, 15.3] | 7.5 [6.0, 9.3] | 2.7 [1.7, 4.1] |
| 25–34 | 3.3 [2.5, 4.4] | 4.1 [3.3, 5.2] | 3.4 [2.6, 4.6] | 9.9 [8.5, 11.5] | 9.6 [8.1, 11.3] | 3.1 [2.4, 4.1] |
| 35 and above | 3.4 [1.9, 5.9] | 6.7 [4.5, 9.8] | 6.3 [3.7, 10.4] | 12.3 [9.5, 15.9] | 7.5 [5.3, 10.5] | 3.5 [2.0, 6.2] |
| **Maternal occupation** |  |  |  |  |  |  |
| Currently working | 3.4 [2.2, 5.1] | 4.4 [3.2, 6.1] | 3.6 [2.5, 5.1] | 11.0 [8.9, 13.5] | 8.8 [7.0, 10.9] | 3.0 [2.0, 4.5] |
| Currently not working | 2.9 [2.3, 3.6] | 4.9 [4.0, 5.8] | 3.3 [2.6, 4.2] | 10.7 [9.5, 12.0] | 8.8 [7.7, 10.2] | 3.0 [2.4, 3.7] |
| **Maternal attitude toward wife-beating** |  |  |  |  |  |  |
| Justified | 2.7 [1.5, 4.9] | 5.6 [3.7, 8.4] | 4.1 [2.2, 7.6] | 14.0 [10.8, 17.8]* | 8.3 [6.2, 11.1] | 3.2 [1.8, 5.6] |
| Not justified | 3.0 [2.4, 3.7] | 4.6 [3.9, 5.5] | 3.3 [2.6, 4.0] | 10.3 [9.2, 11.5] | 8.9 [7.8, 10.2] | 2.9 [2.4, 3.7] |
| **Maternal decision-making autonomy** |  |  |  |  |  |  |
| Practiced | 3.0 [2.4, 3.7] | 4.7 [3.9, 5.6] | 3.1 [2.5, 3.9]* | 10.7 [9.5, 11.9] | 8.7 [7.6, 9.9] | 3.1 [2.5, 3.8] |
| Not practiced | 3.0 [1.9, 4.6] | 4.9 [3.4, 7.1] | 4.9 [3.3, 7.3] | 11.3 [8.9, 14.4] | 9.7 [7.5, 12.5] | 2.4 [1.4, 4.1] |
| **Maternal education** |  |  |  |  |  |  |
| No education | 2.5 [1.0, 6.0] | 8.5 [5.2, 13.6] | 11.2 [7.1, 17.2]*** | 20.5 [15.4, 26.7]*** | 8.4 [5.5, 12.7] | 3.1 [1.5, 6.2] |
| Primary | 2.0 [1.2, 3.3] | 4.5 [3.2, 6.4] | 4.2 [2.8, 6.0] | 14.9 [12.6, 17.7] | 10.2 [8.3, 12.5] | 3.0 [2.1, 4.4] |
| Secondary | 3.1 [2.4, 4.0] | 4.7 [3.8, 5.9] | 2.6 [1.9, 3.6] | 10.1 [8.8, 11.6] | 8.8 [7.5, 10.5] | 3.1 [2.3, 4.1] |
| Higher | 4.1 [2.5, 6.5] | 3.8 [2.5, 5.7] | 2.2 [1.2, 3.9] | 4.5 [3.0, 6.5] | 7.2 [5.1, 10.2] | 2.5 [1.4, 4.5] |
| **ANC visit (*n* = 2,298)** |  |  |  |  |  |  |
| <4 | 3.2 [2.2, 4.6] | 5.5 [4.3, 7.0]** | 3.4 [2.5, 4.6]*** | 11.6 [9.9, 13.6]*** | 9.6 [8.0, 11.6] | 2.0 [1.3, 3.0] |
| ≥4 | 3.1 [2.2, 4.4] | 3.0 [2.1, 4.3] | 1.0 [0.6, 1.9] | 6.4 [5.0, 8.2] | 11.1 [8.7, 14.0] | 2.9 [1.8, 4.6] |
| **Maternal stature** |  |  |  |  |  |  |
| Short (< 150 cm) | 2.5 [1.7, 3.6] | 4.9 [3.7, 6.3] | 4.7 [3.5, 6.2]** | 16.5 [14.5, 18.6]*** | 12.3 [10.4, 14.5]*** | 3.7 [2.7, 5.1] |
| Tall (≥ 150 cm) | 3.3 [2.6, 4.1] | 4.7 [3.8, 5.6] | 2.6 [2.0, 3.5] | 7.5 [6.4, 8.7] | 6.8 [5.6, 8.2] | 2.6 [1.9, 3.4] |
| **Maternal underweight** |  |  |  |  |  |  |
| No | 2.8 [2.2, 3.5] | 4.3 [3.6, 5.2]** | 3.0 [2.4, 3.8]** | 10.2 [9.1, 11.4]** | 8.7 [7.6, 10.0] | 3.0 [2.4, 3.8] |
| Yes | 4.3 [2.7, 6.7] | 7.5 [5.3, 10.4] | 5.9 [3.9, 8.7] | 14.9 [11.7, 18.6] | 9.4 [6.9, 12.8] | 2.8 [1.6, 4.9] |
| **Father's education** |  |  |  |  |  |  |
| No education | 2.7 [1.6, 4.6]* | 5.5 [3.8, 8.0] | 5.3 [3.5, 7.8]* | 16.4 [13.4, 19.9]*** | 10.2 [7.9, 13.0] | 3.9 [2.5, 6.1] |
| Primary | 2.2 [1.5, 3.2] | 4.6 [3.5, 6.2] | 3.5 [2.6, 4.8] | 13.2 [11.3, 15.5] | 9.4 [7.8, 11.4] | 3.0 [2.0, 4.2] |
| Secondary | 2.8 [1.9, 4.0] | 4.7 [3.5, 6.1] | 3.1 [2.2, 4.5] | 9.9 [8.4, 11.7] | 8.5 [6.9, 10.5] | 2.1 [1.4, 3.2] |
| Higher | 5.0 [3.4, 7.2] | 4.4 [3.0, 6.3] | 2.1 [1.2, 3.9] | 3.8 [2.6, 5.6] | 7.3 [5.3, 9.9] | 3.9 [2.5, 6.1] |
| **Exposed with media** |  |  |  |  |  |  |
| Exposed | 3.3 [2.5, 4.3] | 4.9 [3.8, 6.2] | 2.8 [2.0, 3.7] | 8.0 [6.8, 9.3]*** | 8.8 [7.3, 10.5] | 3.2 [2.3, 4.4] |
| Not at all | 2.7 [2.0, 3.7] | 4.6 [3.7, 5.6] | 3.9 [2.9, 5.2] | 13.2 [11.7, 14.9] | 8.9 [7.6, 10.4] | 2.8 [2.1, 3.7] |
| **Toilet facility** |  |  |  |  |  |  |
| Improved | 3.2 [2.6, 4.0] | 4.8 [4.0, 5.7] | 2.8 [2.2, 3.6]*** | 9.8 [8.7, 11.0]*** | 8.3 [7.2, 9.6] | 3.0 [2.4, 3.7] |
| Unimproved | 2.0 [0.9, 4.0] | 4.5 [3.1, 6.4] | 6.0 [4.1, 8.7] | 15.3 [12.5, 18.6] | 11.2 [8.9, 14.0] | 2.9 [1.8, 4.6] |
| **Wealth index** |  |  |  |  |  |  |
| Poorest | 2.3 [1.3, 4.1] | 5.3 [3.9, 7.1] | 6 .0[4.5, 8.1]*** | 17.6 [15.0, 20.6]*** | 10.3 [8.3, 12.7] | 3.6 [2.4, 5.2] |
| Poorer | 2.4 [1.5, 3.7] | 6.0 [4.4, 8.3] | 3.4 [2.3, 5.0] | 12.9 [10.5, 15.9] | 10.1 [7.9, 12.8] | 3.0 [2.0, 4.6] |
| Middle | 2.7 [1.7, 4.3] | 4.8 [3.4, 6.8] | 2.7 [1.7, 4.1] | 10.9 [8.9, 13.4] | 7.7 [5.9, 9.9] | 2.6 [1.6, 4.1] |
| Richer | 3.3 [2.1, 5.2] | 3.3 [2.2, 4.9] | 3.1 [1.9, 5.0] | 6.2 [4.6, 8.2] | 7.8 [5.9, 10.2] | 3.1 [1.9, 4.8] |
| Richest | 4.3 [3.0, 6.1] | 4.1 [2.7, 6.4] | 1.6 [0.8, 3.1] | 5.7 [4.0, 8.0] | 8.3 [5.7, 11.8] | 2.6 [1.5, 4.7] |
| **Place of residence** |  |  |  |  |  |  |
| Urban | 3.5 [2.5, 4.8] | 4.1 [2.9, 6.0] | 3.2 [2.2, 4.8] | 8.7 [7.0, 10.7]* | 9.2 [6.9, 12.1] | 3.5 [2.3, 5.3] |
| Rural | 2.8 [2.2, 3.6] | 4.9 [4.2, 5.9] | 3.4 [2.7, 4.4] | 11.5 [10.3, 12.9] | 8.7 [7.6, 9.9] | 2.8 [2.2, 3.5] |
| **Division** |  |  |  |  |  |  |
| Barisal | 2.7 [1.6, 4.5] | 6.5 [4.4, 9.5] | 4.5 [2.7, 7.5] | 11.2 [8.4, 14.6]*** | 9.4 [6.7, 12.9] | 3.0 [1.7, 5.3] |
| Chattogram | 3.4 [2.1, 5.5] | 4.1 [2.8, 5.8] | 4 [2.4, 6.5] | 12.1 [9.4, 15.3] | 7.9 [5.9, 10.4] | 2.7 [1.6, 4.4] |
| Dhaka | 2.6 [1.6, 4.3] | 3.4 [2.1, 5.5] | 2.5 [1.3, 4.6] | 8.6 [6.5, 11.3] | 9.3 [6.7, 12.7] | 3.5 [2.2, 5.6] |
| Khulna | 2.5 [1.3, 5.1] | 5.4 [3.5, 8.2] | 2.4 [1.2, 4.5] | 8.2 [6.0, 11.2] | 8.5 [6.3, 11.4] | 1.6 [0.7, 3.4] |
| Mymensingh | 3.3 [2.1, 5.3] | 5.7 [4.0, 8.1] | 4.7 [3.1, 7.1] | 12.0 [9.9, 14.4] | 10.8 [8.2, 14.1] | 3.4 [2.2, 5.3] |
| Rajshahi | 2.9 [1.4, 5.7] | 6.1 [3.9, 9.4] | 3.2 [1.7, 5.9] | 8.1 [5.9, 11.0] | 8.6 [5.9, 12.4] | 1.8 [0.7, 4.5] |
| Rangpur | 2.8 [1.6, 4.7] | 6.0 [3.9, 9.0] | 2.7 [1.5, 5.0] | 11.1 [8.5, 14.4] | 8.1 [5.4, 11.9] | 4.1 [2.5, 6.6] |
| Sylhet | 3.9 [2.5, 6.0] | 3.8 [2.2, 6.4] | 4.4 [2.8, 7.0] | 18.6 [15.0, 22.8] | 9.2 [6.7, 12.5] | 3.6 [2.2, 5.9] |

*P value<0.05, **P value<0.01, ***P value<0.001
